# Home-Based Connected Devices Combined With Statistical Process Control for the Early Detection of Respiratory Exacerbations by Patients With Cystic Fibrosis: Pilot Interventional Study With a Pre-Post Design

**DOI:** 10.2196/51753

**Published:** 2024-10-28

**Authors:** Enora Le Roux, Moreno Ursino, Ivana Milovanovic, Paul Picq, Jeremie Haignere, Gilles Rault, Dominique Pougheon Bertrand, Corinne Alberti

**Affiliations:** 1 Inserm Hôpital Universitaire Robert Debré Assistance publique – Hôpitaux de Paris.Nord - Université Paris Cité Paris France; 2 Epidémiologie clinique-évaluation économique appliqué aux populations vulnérables Inserm Université Paris Cité Paris France; 3 Inserm Centre de Recherche des Cordeliers, Sorbonne Université Université Paris Cité Paris France; 4 Laboratoire Educations et Promotion de la santé Université Sorbonne Paris Nord Villetaneuse France

**Keywords:** connected devices, cystic fibrosis, patient education, self-management, medical device, home monitoring, remote monitoring, statistical process control, connected health, alerts

## Abstract

**Background:**

Currently, patients with cystic fibrosis do not routinely monitor their respiratory function at home.

**Objective:**

This study aims to assess the clinical validity of using different connected health devices at home to measure 5 physiological parameters to help prevent exacerbations on a personalized basis from the perspective of patient empowerment.

**Methods:**

A multicenter interventional pilot study including 36 patients was conducted. Statistical process control—the cumulative sum control chart (CUSUM)—was used with connected health device measures with the objective of sending patients alerts at a relevant time in order to identify their individual risk of exacerbations. Associated patient education was delivered. Quantitative and qualitative data were collected.

**Results:**

One-half (18/36) of the patients completed the protocol through the end of the study. During the 12-month intervention, 6162 measures were collected with connected health devices, 387 alerts were sent, and 33 exacerbations were reported. The precision of alerts to detect exacerbations was weak for all parameters, which may be partly related to the low compliance of patients with the measurements. However, a decrease in the median number of exacerbations from 12 months before the study to after the 12-month intervention was observed for patients.

**Conclusions:**

The use of connected health devices associated with statistical process control showed that it was not acceptable for all patients, especially because of the burden related to measurements. However, the results suggest that it may be promising, after adaptations, for early identification and better management of exacerbations.

**Trial Registration:**

ClinicalTrials.gov NCT03304028; https://clinicaltrials.gov/study/NCT03304028

## Introduction

### Background

Respiratory exacerbations are the major cause of decline in respiratory function, which is the main cause of death, in patients with cystic fibrosis [1]. An early diagnosis of exacerbations is recommended for effective treatment and to avoid a decline in respiratory function [2]. Identifying the warning signs of exacerbations is therefore a priority.

Currently, patients with cystic fibrosis do not routinely monitor their respiratory function nor the associated physiological parameters at home [3].

Therefore, exacerbation episodes may be diagnosed late, as symptoms progress, or when the patient is required to consult his or her doctor. The development of an effective approach to detect early indicators of exacerbations using home monitoring should be explored.

Several studies of the daily monitoring of exacerbation symptoms in patients with cystic fibrosis have been conducted. Most of these studies have focused on only one parameter of respiratory function, sometimes accompanied by an isolated reporting of symptoms or in the form of a symptom score [4,5]. However, there are numerous indicators of pulmonary exacerbation in cystic fibrosis, and these studies did not intentionally choose the most relevant indicator [6].

Furthermore, no study involved a patient empowerment perspective since the monitored data were transferred to the medical staff for decisions about how to adapt care. However, patient participation in decisions is important to allow them to have greater control of their health, and home-based monitoring via connected tools could allow this [7].

Finally, the use of personalized alert thresholds for the different physiological parameters, as measured by each patient, has never been tested, although it is known that the signs of exacerbations differ from one patient to another.

### Hypothesis and Objectives

Our hypothesis was that the use of statistical process control is promising for monitoring indicators and allows early detection of exacerbations by patients and rapid identification of variations in these signs using control charts. This study aimed to assess the clinical validity of the use of different connected health devices at home combined with alerts sent to patients who received adequate education, based on individualized thresholds for early exacerbation detection and without data transmission to the care professionals. Acceptability (subjective experience, compliance with measurements) as well as the evolution of the patients’ health status were also measured.

## Methods

### Trial Design

**Figure 1:**
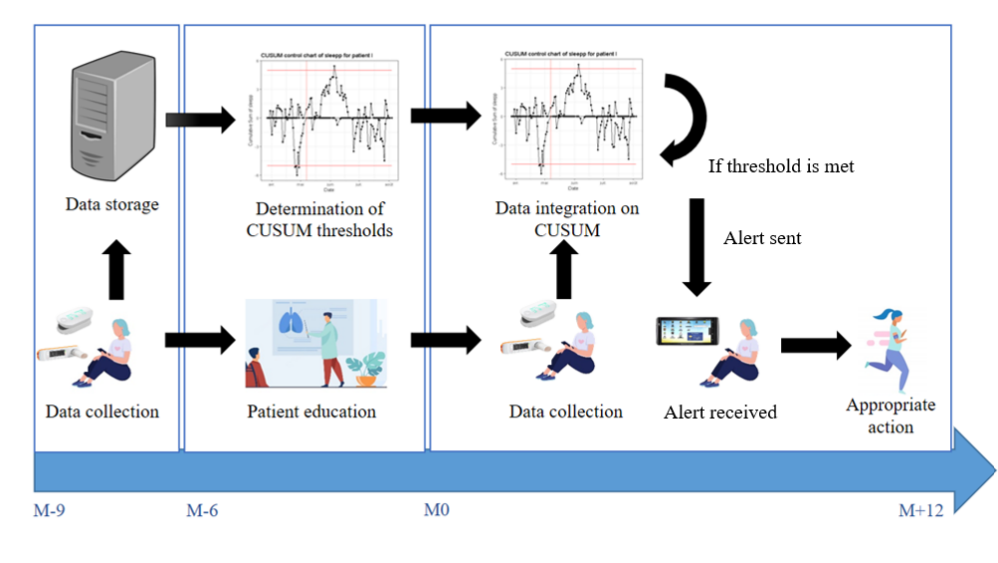
Study timeline. CUSUM: cumulative sum control chart.

**Figure 2:**
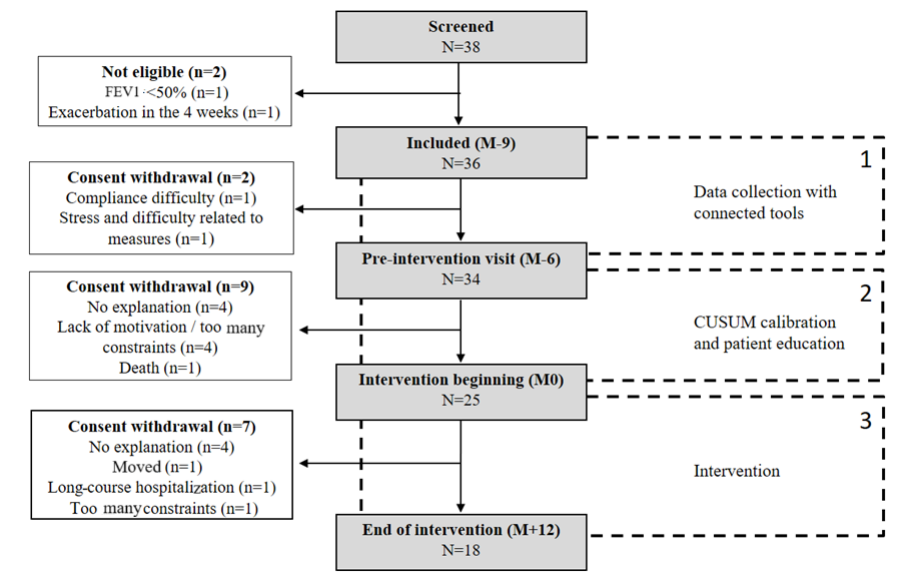
Participant flow and recruitment. CUSUM: cumulative sum control chart; FEV1: forced expiratory volume in 1 second.

**Figure 3:**
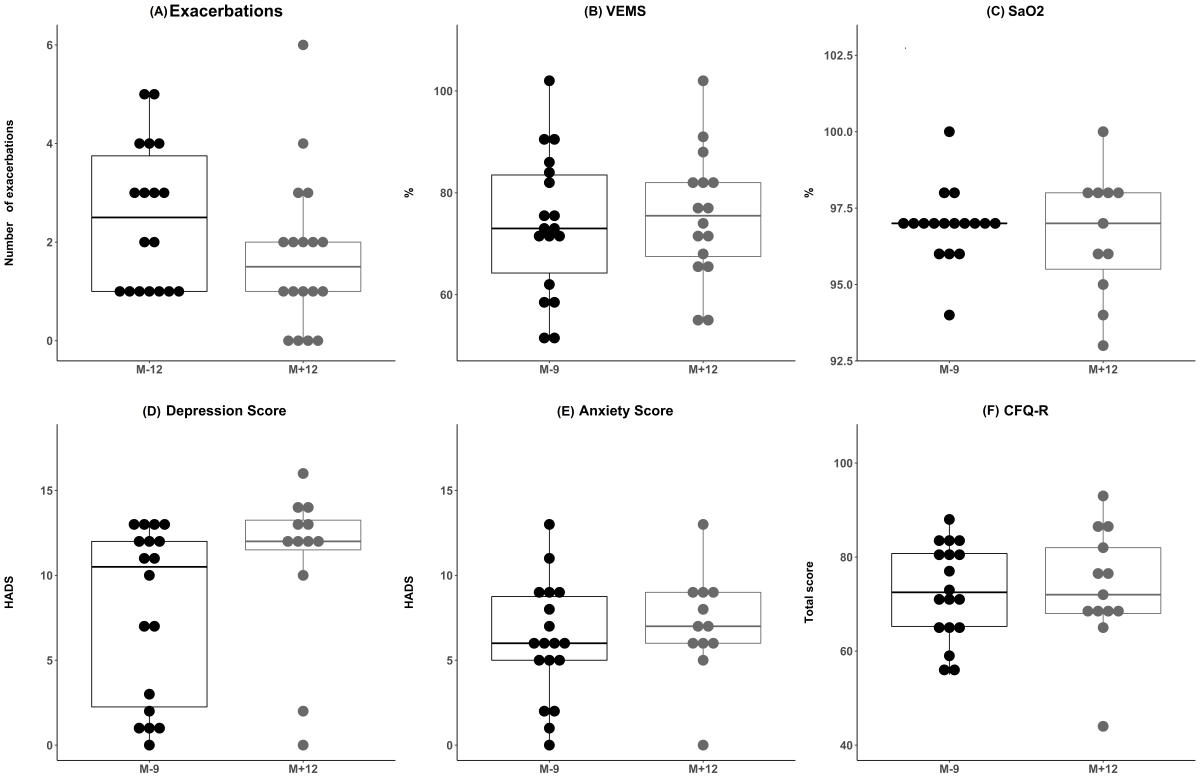
Box plots showing the median values and first and third quartiles for (A) exacerbations, (B) forced expiratory volume in 1 second (FEV1), (C) oxygen saturation (SaO2), (D) Hospital Anxiety and Depression Scale (HADS) depression score, (E) HADS anxiety score, and (F) Cystic Fibrosis Questionnaire-Revised (CFQ-R) score before (M–9 or M–12) and after (M+12) the intervention, which started at M0, for the patient subgroup who made the visit at M+12 (N=18).

A nonrandomized, interventional, multicenter study was conducted. This was a pilot study examining the feasibility and relevance of our approach before considering a larger scale study. Patient participation involved 3 steps: (1) initial intensive data collection using connected health devices (3 times per week for 3 months; M–9 to M–6; Figure 1); (2) determination of individualized thresholds based on the patients’ baseline data and patient education (6 months to 8 months; M–6 to M–0; Figure 1); (3) intervention and follow-up (12 months; M0 to M+12; Figure 1). Details of the intervention timeline are available in the study protocol [8]. A qualitative study is included in the protocol. The qualitative results from the patient education are presented elsewhere [9], while the patients’ feelings regarding acceptability are presented in this article.

The study is reported according to the CONSORT 2010 checklist (Multimedia Appendix 1) [10].

### Participants

#### Eligibility Criteria for Participants

Patients older than 12 years, who had a forced expiratory volume in 1 second (FEV_1_) >50%, who were in stable clinical condition at the time of inclusion, who had at least one exacerbation in the previous year, who had a long-term prescription for pulmonary symptoms, with WiFi access at home, and who gave their consent or whose parents gave their consent (if the patient was a minor) were included in this study.

#### Identification of Participants and Consent

Patients were recruited from 7 pediatric or adult hospitals with expertise in cystic fibrosis in France. Centers were chosen based on their motivation to participate and their diversity. They are located in 5 different regions of France, reaching a diverse population of patients (age, urban/rural areas, socioeconomic characteristics).

Hospital staff emailed all potentially eligible patients or parents (if minors) to present the study to them and ask them to participate. According to the email responses, study staff then telephoned the patients or parents who wished to participate. They checked their eligibility, gave them additional information, and answered their questions. The inclusion visit was then scheduled for the next hospital visit.

### Intervention

The intervention consisted of the home use of connected devices integrating the cumulative sum control chart (CUSUM) tool for the monitoring of indicators in order to detect signs of exacerbation early in patients with cystic fibrosis.

### Connected Tools and Measures

The process of selecting the parameters to measure and related connected tools to use is detailed in the protocol [8]. Briefly, based on the results of a consensus study [6], indicators to be measured by connected tools were identified, and connected tools capable of collecting these relevant parameters with a focus on exacerbation detection were selected through market analysis. We selected 5 devices from 2 French companies (Lamirau and Withings).

Each patient was equipped with a spirometer, an oximeter, a weight scale, a watch (with a pedometer option), and a lamp connected with sensors placed under the mattress. These devices measure FEV_1_, heart rate, oxygen saturation, weight (standard deviation), sleep duration (minutes/night), and physical activity (step count/day). A 7-question yes/no questionnaire was completed for spirometer use; these questions of perception (on cough, fatigue, physiotherapy, appetite) were not used by the CUSUM but allowed the patient to review the onset of certain symptoms and their relationships with the variations in the measured parameters for educational purposes.

Tablets with the relevant apps were provided to the patients for the research period. During the inclusion visit, the devices were connected to the tablets, the clinical research team demonstrated the use of the devices for the patients, and the patients received written instructions for the use of the connected devices and maintenance support. An email address specific to the study (different from their usual email address) was provided to the patients to connect to the various applications from the tablet provided as part of this study and to receive alerts in case of abnormal measurement values. This was planned to ensure data security and guarantee separation between personal data and research data. The email address did not allow patient identification (we did not use the usual firstname.lastname@domain.fr but a non-identifiant-code@domain.fr).

### CUSUM

The CUSUM control charts reported the variation in the measured indicators and signaled the moment when an anomaly appeared in a process. These charts are widely used in the field of manufacturing production and have recently become popular in the medical field [11].

The principle of control charts is based on graphs in which an indicator is monitored over time. The graph includes defined limits of “conformity,” called lower and upper control limits, which are bounds within which the process is said to be “under control” [12]. In addition, control charts can help determine whether these changes are linked to a specific cause or simply due to natural variability [13]. This appears to be very practical for the detection of adverse health events [14]. It therefore seemed suitable for monitoring indicators of exacerbations.

Based on the first 3-month period of intensive data collection by patients, one CUSUM chart to monitor each indicator for each patient was developed. Thresholds were defined based on variations observed during the periods without an exacerbation in this first period. Clinical teams were involved in the definition and modification of patients’ thresholds (if needed, such as when the predefined thresholds were too sensitive to change, which resulted in the sending of very frequent and unjustified alerts). When one indicator, as measured by the patient using the connected tools, exceeded the limit, an alert was sent to the patient’s study email address. The personalization of the thresholds was necessary because there was great variability in the expression of the indicators among patients. The control charts (visual representation) facilitated discussions with the clinicians. Revision of the thresholds took into account clinical events that could explain the variations, and the parameters were not looked at independently from each other but all together for greater relevance.

Ideally, CUSUM validity and interpretability are assured when measures are taken regularly since CUSUM belongs to the time series method. The patients were asked to perform 2 to 3 measurements per week with the aim of identifying exacerbations as early as possible. This frequency was also chosen in a similar protocol [15].

### Patient Education

An educational program entitled “React to the warning signs of an exacerbation” was delivered to all patients before alerts were sent [16]. It taught them how to understand and react to the measures and alerts according to the personalized action plan set up with their clinician during the education and according to their own resources.

### Outcomes

The main outcome, clinical validity, was assessed by measuring the agreement (using precision calculation) between the episodes of exacerbations detected by the connected devices taking the form of alerts and the episodes of exacerbations (+/–3 days) identified by the start date of the antibiotic treatment reported in the patient’s medical file.

The secondary evaluation criteria were those related to (1) protocol acceptability, including compliance with the use of connected devices at home (number of measures of each indicator during the entire period study) and continued participation according to the protocol, and the subjective participant experience collected at the end of the follow-up (after M+12) using semidirective interviews and (2) the evolution of patient-reported outcomes and physiological outcomes measured during medical consultations between M–12 and M0 (before the intervention) and between M0 and M+12 (during the intervention; Figure 1), number of exacerbations between M–9 and M+12, FEV_1_, weight (standard deviation), patient anxiety and depression measured using the Hospital Anxiety and Depression Scale (HADS), and patient quality of life assessed using the Cystic Fibrosis Questionnaire-Revised (CFQ-R) scale.

### Sample Size

This pilot study was exploratory, and the number of patients enrolled was based on the recruitment capacity of the participating centers over the period of the study. Given the constraints related to study management at each center as well as the saturation threshold for the qualitative study, which is usually set at 30 patients, a 20% dropout rate was anticipated and justifies the initial recruitment of 36 patients.

### Data Collection

A high level of protection and security was ensured for participants’ personal data during data collection and management. Given the complexity of the data transmission, the data management plan was developed by a multidisciplinary team including representatives from connected device companies, IT specialists, a data manager, a quality manager, and a representative from the regulatory aspect. Data collected from medical records and study questionnaires were reported by the investigators or their representatives in an electronic case report (Cleanweb) form hosted on a secure server managed by the research team. Transmission of the data collected by the connected devices involved several steps: Connected devices sent data to the tablet delivered to the patients for research when the devices were synchronized after a measurement. These data were sent to the connected device companies according to the usual transmission pathway for ordinary users. Access to these data was allowed for the research team via the companies’ applications. The data were transferred to the secure server managed by the research team through a secure website. Only the data necessary for the search were transferred. The state of data collection from connected objects was checked regularly; the source missing data or data inconsistencies was sought throughout the study.

### Statistical Methods

Quantitative variables are described using their median (1st quartile-3rd quartile) and minimum and maximum values. Categorical variables are described using their numbers and percentages. Regarding the descriptive statistics, patients were classified into 2 groups: those who partially or fully participated in the intervention (between M0 and M12) and those who did not (dropped out between M–9 and M0).

To compute the precision of the 5 selected indicators, for each alert sent, a binary variable was generated with the value of 1 if the alert was sent within +/–3 days of an exacerbation. The precision was then defined as the sum of the alerts sent within the window (+/–3 days of an exacerbation) divided by the total number of alerts sent for the same indicator.

The secondary criteria were assessed, using box plots, in the subgroup of patients who made the visit at M+12.

Statistical analyses were performed using the SAS Statistical Package, version 9.4 (SAS Institute Inc) and R version 4.1.1 (2021-08-10).

### Qualitative Analysis

All patients were asked to participate in qualitative interviews at the end of the protocol. These interviews were conducted by a researcher in public health who specialized in cystic fibrosis and patient education. The interviews were based on a semistructured guide to collect experiences related to the protocol, the use of the tools of the study, and their implications in daily life and care. In the context of this article, the quotes are used strictly for illustrative purposes.

### Ethics Approval

The entire study, including the quantitative and qualitative research, received ethics committee approval (Comité de protection des personnes Nord Ouest III) on June 10, 2017 (#2017-A00723-50).

The written informed consent of all patients (and parents if the patient was a minor) participating in the research was obtained by the physicians who usually provided care to the patients and who participated in the protocol. The information on the research was provided orally and in writing.

## Results

### Participants

From October 20, 2017, to June 18, 2018, 38 patients were screened for inclusion. Before the inclusion visit, 2 patients became ineligible. Thus, 36 participants were included, and 11 patients left the study before the beginning of the intervention (M0; Figure 2).

Table 1 illustrates the demographic and psychometric characteristics of the participants at baseline, by separating those who partially or fully participated in the intervention (M0 to M+12) from those who stopped before (between M–9 and M0). Those who withdraw before the intervention had a tendency to be younger, female, and anxious.

**Table 1:** Demographic and psychometric characteristics of the participants at baseline (M–9).

Variable			Patients who started the intervention (N=25)^a^		Patients who withdrew before the intervention (n=13)^b^
Age (years), median (Q1-Q3)			23.9 (15.2-28.1)		20.3 (15.9-23.8)
Age (years), range			13.2-44.5		12.3-40.3
**Sex, n (%)**					
	Male	14 (56)		3 (23)	
	Female	11 (44)		10 (77)	
Number of exacerbations in the 12 months before inclusion, median (Q1-Q3)			3.0 (1.0-3.0)		3.0 (2.0-3.0)
HADS^c^ depression score ≥11, n (%)			14 (56)		3 (27)^d^
HADS anxiety score ≥11, n (%)			2 (8)		2 (18)^d^
Total CFQ-R^e^ score, median (Q1-Q3)			77.0 (68.0-83.0)^f^		77.0 (63.5-80.0)^g^
Total CFQ-R score, range			64.0-88.0^f^		51.0-82.0^g^

^a^Partially or fully participated in the 12-month intervention.

^b^Stopped participating in the 9 months before the intervention started.

^c^HADS: Hospital Anxiety and Depression Scale.

^d^Data missing for 2 participants.

^e^CFQ-R: Cystic Fibrosis Questionnaire-Revised.

^f^Data missing for 13 participants.

^g^Data missing for 5 participants.

### Clinical Validity

During the intervention, 33 exacerbations were reported by the patients who participated in the intervention phase. During the 12-month intervention, 387 alerts were sent. The alert precision was weak (Table 2).

**Table 2:** Precision of the 387 alerts sent during the 12-month intervention.

Indicator	Alerts sent, n	Exacerbations during the intervention period (+/–3 days), n	Precision
Heart rate	121	4	0.033
Weight	43	0	0
SaO_2_^a^	70	3	0.043
Sleep	102	0	0
FEV_1_^b^	51	0	0

^a^SaO_2_: oxygen saturation.

^b^FEV_1_: forced expiratory volume in 1 second.

Some alerts that should have been sent were not, mainly because of a computer server bug, uninform database updates by the connected device manufacturers, and problems with synchronization with the connected objects (failure in the Bluetooth or WiFi network at patients’ homes). A sensitivity analysis of the alerts that should have been sent showed no difference in the precision performance.

### Protocol Acceptability

Over the entire intervention period, patients completed 6162 measurements. Compliance with the measurements differed according to the measured parameters (Figure S1 in Multimedia Appendix 2). The majority of the patients completed their measures for 17 weeks to 20 weeks postintervention but discontinued after that.

We identified 5 patient profiles according to their compliance with the measurements (Figure S2 in Multimedia Appendix 2): (1) very compliant before M0 and during the intervention for all parameters (n=3), (2) very compliant before M0 and compliant with a selection of parameters during the intervention (n=5), (3) very compliant before M0 and moderately compliant during the intervention for any parameter (n=4), (4) compliant before M0 and not compliant during the intervention (n=8), (5) not compliant before M0 nor during the intervention (n=5). Over time, compliance differed by parameter, patient preference, and deterioration in the device’s operation. Sleep was one of the most measured parameters. Some patients only completed measurements very occasionally. Feedback from the patients indicated that these measurements were used to monitor the evolution of their exacerbations when they suspected an exacerbation was occurring or to reassure patients when their body signals worried them.

For each patient profile, illustrative quotes were selected to better understand the elements that acted in favor or against the completion of measurements (Table 3).

**Table 3:** Compliant patient profiles and reasons cited for the evolution of compliance.

Profiles	Description	Quotes
1	Family support (this was particularly observed for adolescents supported by their parents), being comfortable with the connected health devices, and including the measures in a therapeutic routine were reported by these patients.	“[the measurement] it was regular, 3-4 times a week. It took time, but anyway the treatment I had I was doing it at the same time [...] it was quite easy to use. My mother was watching with me [...] Gradually, I really got involved.” [Participant #8]
2	The failure of some connected health devices to produce reliable data, the distress of some patients facing the results of their measures alone at home, and the difficult balance between the frequency of measures to perform and their potential impact on personal life were reported by these patients. A choice of tools was made by some patients according to their perceived relevance, acceptability, or ease of use.	[after a measurement done alone at home which was abnormally “bad” on the spirometer] “I didn't want to do it again alone. I didn't feel capable of it at all, and so I brought my device [to the hospital], and I said: Could we recalibrate it together? And in fact it was too complicated to recalibrate it, because...I don't know, the wifi wasn't working, or...they didn't have the time, they didn't have the possibility to do it, so I left with my device, and I never had the courage to do it again. [...] [before that] I didn't do it three times a week, I did it every day, and even three times a day in times of superinfection [...] it started to take too much space in my life, in fact.” [Participant #15]
3	Use of the tools evolved over time for these patients. In the preintervention period, the patients observed that their measures changed during exacerbations; over time, some of them only completed their measurements to reinforce when they suspected an exacerbation rather than as a routine measure	“It really depends on the need. [...] during exacerbations, well, I tend to use them every day. [...] I would like to continue using them regularly. [...] today, I tend to call the hospital more quickly, to redo the measurements to see which parameter is wrong, and to inform the hospital...” [Participant #25]
4	The number of devices, lack of support from the hospital teams, anxiety related with certain measurements, technical bugs, and limits of the reliability of the measurements resulted in decreased motivation for these patients to complete measurements over time.	“at one point I was no longer very, very observant, but because it's not very pleasant to see the results...how to say...fall [...] I think there was also the effect of novelty, and then I was doing pretty well the first three months, so I really followed it to the letter. And then [...] there was a given moment when the idea of...just the idea, in fact, of taking measurements with the connected devices, could cause anxiety. [...] there were also a lot of technical issues, and there was a time, in fact, when I no longer trusted the results I had in front of me. [...] it seems to me that the [hospital] teams did not have feedback on the data so they could not really know that I was not doing it. But it's true that I think I felt a little abandoned at some point.” [Participant #13]
5	The lack of added value of the measurements made by the devices in relation to the patients’ own perceptions, the technical problems, and weaknesses of the tools chosen for the protocol explained the lack of involvement of these patients in the measurements.	“me personally, when I start to have exacerbations, I already notice it very quickly, since I feel that I am coughing very quickly abnormally [...] it is true that connected objects did not work for very very long unfortunately [...] in terms of anticipation, it's true that I know my body quite well, so I manage to anticipate very easily. [...] maybe connected objects weren't the best fit either, let’s say.” [Participant #16]

### Subjective Experience

It was not expected that deleterious effects would be observed in this protocol. The qualitative interviews, however, highlighted some, like the anguish generated by certain measures due to the difficult balance between the number of measurements to be carried out and mental well-being.

Of course, when everything is going well, it's easy to use connected objects, and then when things start to go a little worse [...] when you can't find an effective treatment. It's already a situation that can be a source of anxiety, and suddenly, at that time, I found that connected objects added anxiety.

Participant #4

On the other hand, in the qualitative interviews, the patients reported general satisfaction with their participation in the protocol (regardless of their compliance profile), better knowledge of themselves, and for some, the relevance of using these devices during COVID-19.

I had an upsurge in the use of objects, during lockdown, because I have one of my appointments which was canceled [...] It was a fairly positive experience, even if it caused me anxiety at times, I find that it still allowed me to be very, very aware of my body [...] to understand that my body was not against me but with me, and that the signs it was sending me were actually for me, and that they were signs to take into account to take care of me.

Participant #13

The security procedures implemented by the research team, in particular for consulting the alerts, hindered the use of these for some patients (and their parents in the case of minors).

It is not the child who will check the emails, and in terms of patient education and autonomy in care, we are missing something that could be done by having the right format...the good mode of communication with children [...] And even adults. Because me, personally, if I had cystic fibrosis, I would prefer to receive an alert on my smartphone in real time or on my watch.

Participant #15

The methodological choice of the CUSUM, implying that the rules for sending alerts were not based on exceeding absolute values ​​but on the accumulation of variations in a measurement, also caused difficulties in understanding alerts, which could affect patient empowerment.

What seems strange to me is that often I am no longer at 95-96, I have even reached 98-99 sometimes, it happened to me the last time, and at these times I do not receive any alert. On the other hand, at 97, I receive an alert.

Participant #7

### Effect on Patients’ Health Outcomes

For the patients who participated in the intervention, we observed a decrease in the median number of exacerbations (from 2.5 to 1.5) from the 12 months before inclusion in the study to the 12-month intervention. For the secondary outcomes, we observed an increase in the depression score. No other changes were observed (Figure 3). For the values shown in Figure 3, the median FEV_1_ values were 73% and 75.5% (data missing for 2 patients) at M–9 and M+12, respectively. Median oxygen saturation values were 97% at both M–9 (data missing for 1 patient) and M+12 (data missing for 7 patients). The median HADS depression scores were 10.5 and 12 at M–9 and M+12 (data missing for 6 patients), respectively, and the median HADS anxiety scores were 6 and 7 at M–9 and M+12 (data missing for 6 patients), respectively. Finally, the median CFQ-R scores were 72.5 and 72 at M–9 and M+12 (data missing for 5 patients), respectively.

## Discussion

This study has highlighted salient points concerning the feasibility and relevance of home monitoring coupled to the CUSUM method and patient education in patients with cystic fibrosis.

### Feasibility and Patient Preferences

This study showed that research involving connected tools, to be used routinely and at high frequency with the aim of prevention of exacerbations, did not correspond with the characteristics of all patient profiles. We identified difficulties with long-term compliance according to the different patient profiles, which could be the subject of more detailed analysis to better understand how to promote compliance with home monitoring and by whom. We saw that more anxious patients were not the best suited for this type of protocol.

As in other studies [6,17], adherence to the health monitoring intervention was lower than expected. Barriers to using health-related connected tools have already been reported and include inadequate information, limited usability of the technologies, challenges associated with using multiple health IT, and technical problems [18]. These were also observed in this study. Other difficulties were also noted in our study: There were many tools per patient, the research delays meant that some tools were almost obsolete at the time of their use by the patient, replacement or repair of the tools was not planned in advance, and the project team lacked a dedicated technician. These points must be integrated into the protocol of future studies involving connected tools in order to improve their feasibility.

We also observed that the patients did not all have the same preferences regarding the measures they used to monitor their exacerbations. Sleep was one of the most measured parameters and was less distressing than respiratory function measurements. It is a passive measure that makes it a relevant and feasible parameter to integrate into home monitoring. In fact, sleep is known for its link with altered quality of life of patients with cystic fibrosis, and sleep disorders are common among patients with acute exacerbations [19-21].

Patients also had different ways of using the connected tools. Some used them in an exploratory way independent of their perceptions of health (which was how they were asked to use them by the protocol), and others used them in a confirmatory way when their perceptions were altered and they wanted to confirm them using objective data. As part of the protocol, the patients were encouraged to listen to their body (patient education session based on empowerment, perception questionnaires, and measurements). Patients who succeeded at understanding their body signals better during the protocol probably “hijacked” the initial exploratory use of the connected devices with confirmatory use over time.

### Research Team’s Experience: Lessons Learned

The research team responsible for the quantitative component of the study ensured the data transmitted by the connected tools was received correctly, managed the CUSUM process and the sending of alerts, carried out the data management, and analyzed the quantitative data. We observed numerous obstacles to conforming to the protocol. First, in the partnerships with the 2 companies that display the connected devices (that were not pharmaceuticals nor medical devices), there was no personal contact for 1 company to manage problems with data feedback, unexpected updates that disrupted the reporting of research data, unplanned difficulties with delivering devices, and data transmission. Second, the pace at which the devices evolved did not match the research timeline, resulting in the use of tools that had become obsolete or that did not reflect the best devices available on the market at the time of the intervention. One of the companies did not distinguish the research patients from their usual clients, so there was no specific training for patients or professionals involved in the research. Instead, we had to use the “traditional” after-sales services for patients when questions arose or bugs happened, and care and research professionals were de facto involved to solve technical problems and patients’ questions more than expected (and more than budgeted).

### Clinical Relevance

One of the unique features of our work was to use personalized thresholds to send alerts to patients when any parameter exceeded what was considered “normal” for them according to their own basic state. For this, we used the CUSUM tool.

It had several advantages but also significant disadvantages for the feasibility of the study: It was very restrictive for the patients to enter their data as regularly as the CUSUM requested, it required daily checks by the research team, and the interpretation of the graphic maps for the care team to define the thresholds for each patient were not the most understandable.

Moreover, the interviews with patients highlighted that the alerts were nonsystematically used and understood; the absolute value delivered by the health-related connected object was initially useful for the patients. Comprehension of the criteria to send alerts and the logistics of looking at a mailbox specifically dedicated to the study may have complicated the use of alerts. This leads us to believe that CUSUM may not be the most adequate method for this type of research, although the use of individually defined “thresholds” that take into account “normal” variations of patients’ parameters should be incorporated into the methodology, based on our experience.

The results showed that the alerts based on the CUSUM were not sent precisely at the time that exacerbations occurred. There are several explanations for this: The first—the fact that our hypothesis was wrong—is that the CUSUM does not allow detection of exacerbations from the parameters measured, the second is that the “validity” of the CUSUM tool may have been limited by the absence of sufficiently repeated patient measurements, and the third is that the alerts allowed patients to adjust their behaviors and avoid exacerbations. In fact, exacerbations in this protocol were defined by the initiation of antibiotic therapy, but, as part of the patient education delivered in the study, patients learned to react to alerts (eg, intensifying physiotherapy, increasing their physical activity). Therefore, it is possible that the alerts allowed patients to avoid initiation of antibiotic therapy. This seems possible given the comparison of the number of exacerbations before and after the start of the intervention. We are unable to form a conclusion about that, and other factors may have contributed to the decrease in exacerbations, such as the Hawthorne effect, patient education itself, COVID-19 lockdowns for the patients recruited later in the study, and change in treatments (eg, some patients received cystic fibrosis transmembrane conductance regulator modulator therapies, which can act on respiratory outcomes, during the protocol) [22,23]. In another study of home monitoring of cystic fibrosis, the intervention was associated with an increase in exacerbations that required more oral antibiotics. In their protocol, all data were sent to the professional caregivers [5] who may have worried and reacted more quickly for patients for whom they had frequent data than for those in the control group for whom they did not. Our results were also not consistent with another study that showed an increase in the detection of exacerbations with home monitoring [17]. This difference may be explained by the absence of associated, adapted patient education to react to data. In another study based on a smartphone app for reporting symptoms by adults with cystic fibrosis, the authors reported a reduction in the number of courses of intravenous antibiotics administered to patients during the 12-month intervention period. Their hypothesis was that the study led to increased contact with the cystic fibrosis center that allowed action before exacerbations deteriorated [24].

The definitions of exacerbations differ, and the associated symptoms vary [25]. In our protocol, the fact that patients chose different health indicators to inform them of their exacerbations supports the need for future protocols to allow for “personalization” of home monitoring measures. The use of connected tools in a larger observational study could allow identification of the relevant indicators to use alone or in combination to detect exacerbations in a variety of patients and advance personalized detection. A study conducted with pediatric patients showed that FEV_1_ combined with the Respiratory Symptom Score could help predict exacerbations in children, but there is substantial uncertainty surrounding the specificity and sensitivity estimations due to the small sample size [14]. However, other methods for early detection of exacerbations have to be developed for patients for whom the acceptability of these tools in daily life is low.

Although daily use of the tools is not recommended for all patients, their use seems promising in the case of difficulties in accessing care, as was the case during lockdown. Home monitoring can allow the patient to be better informed about their state of health to communicate during consultations by videoconference or to better estimate their need for care from time to time [26].

This study also proposed, in an innovative way, that data measured by patients should not be transmitted to health care professionals: We saw advantages in terms of empowerment and disadvantages in terms of motivation and distress when reading the results alone. This phenomenon was also observed when results were reviewed alone but also transmitted to care staff, but surely with a different importance [5]. In other studies in which data were sent to hospital teams, patients reported being reassured that their health was being assessed between clinic visits [5].

### Strengths and Limitations

This study had several strengths: testing different parameters, double the measurements at home, specific patient education, qualitative interviews, being conducted at several sites, involving pediatric and adult patients. However, with regard to the “pilot” aspect of the study, it allowed us to become better informed about the feasibility, but it did not allow us to make conclusions on the effectiveness of the intervention. The absence of a control group and the small number of patients, in addition to numerous dropouts, limit the generalizability of the results.

### Implications

This study offers avenues for the development of future interventional studies using home monitoring with patients with cystic fibrosis and warrants confirmation of the effects obtained regarding the reduction in exacerbations. The implications for progression from the pilot to a future definitive trial consist of the revision of the modalities for the use of connected tools (over a shorter time, less frequent measurements, use the information for educational purposes, identification of key periods when using devices is perceived as relevant for the patients, choice of relevant devices based on patients’ preferences and needs), revision of the definition of thresholds to detect abnormalities (using other methods of statistical control of processes or fixed values ​​by individuals over short periods), and selection of the target population.

### Conclusion

The use of connected health devices associated with statistical process control could be an interesting tool for early identification and better management of exacerbations, but it requires adaptations to be used on a large scale. The protocol was not acceptable for every patient, since it could be perceived as very demanding or anxiety-inducing. However, for patients actively involved in their measurements, we observed a decrease in the median number of exacerbations and an improvement in symptom management and comprehension.

## Appendix

Multimedia Appendix 1 CONSORT 2010 checklist.

Multimedia Appendix 2 Compliance with measurements across the study period and patient profiles regarding device use.

## Abbreviations

CFQ-R: Cystic Fibrosis Questionnaire-Revised
CUSUM: cumulative sum control chart
FEV1: forced expiratory volume in 1 second
HADS: Hospital Anxiety and Depression Scale

## Data Availability

Data are available on request but are subject to the establishment of an agreement due to privacy or other restrictions.

The code used to process the data set (R version 4.4.1 and SAS version 9.4) is available from the corresponding author on reasonable request.
