# Supplementary material for: Home-Based Connected Devices Combined With Statistical Process Control for the Early Detection of Respiratory Exacerbations by Patients With Cystic Fibrosis: Pilot Interventional Study With a Pre-Post Design
Source: JMIR Form Res. 2024 Oct 28;8:e51753. doi: 10.2196/51753 (PMC11555459; doi:10.2196/51753)

**Figure S1.** Compliance with measurements across the study period.


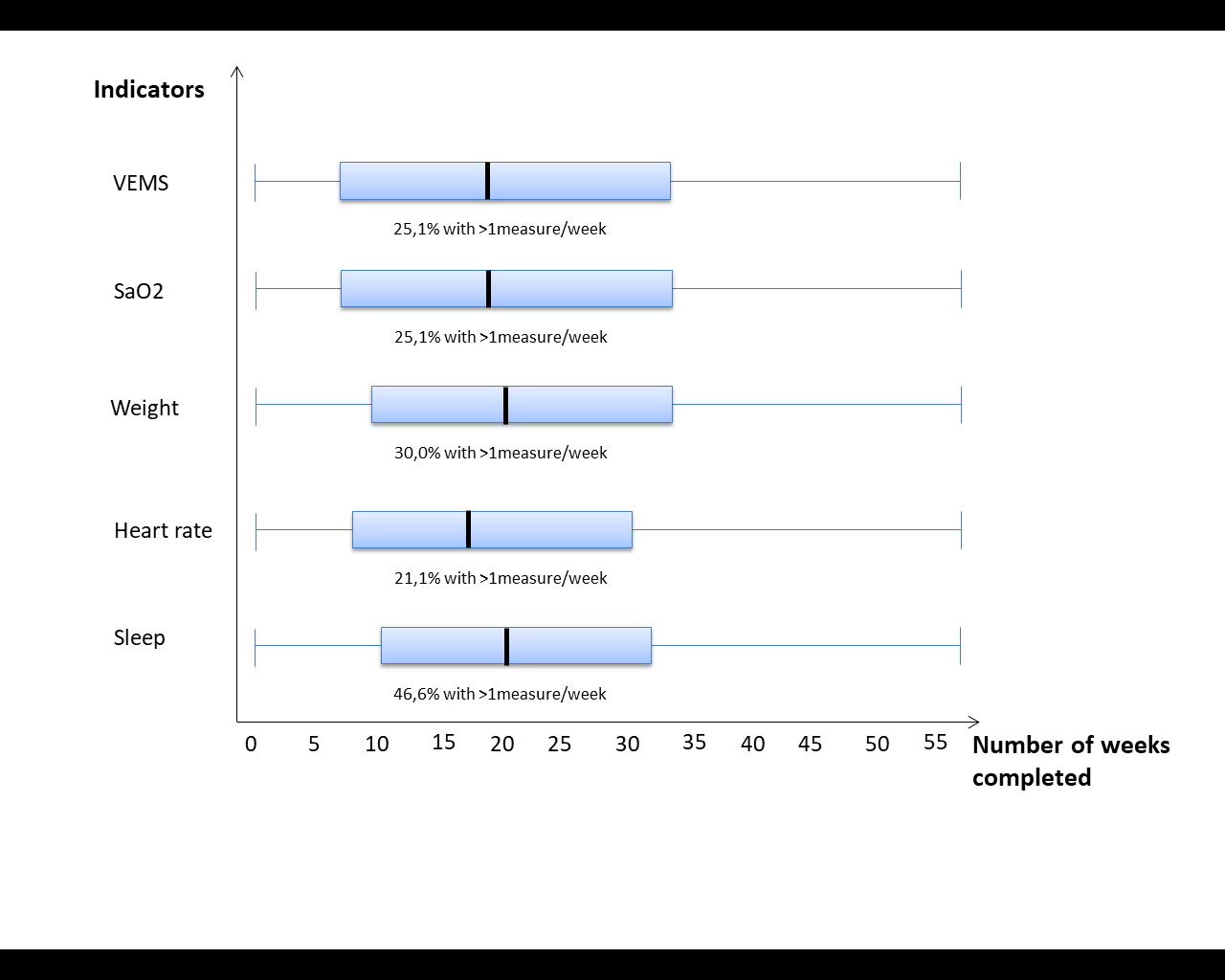


Boxplots show the median value (black bold line in the middle of the box), and the upper and lower outlines of the box correspond to the first and third quartiles. For completeness, the minimum and maximum observed values are represented by the fine vertical lines at the end the lines extending out of the boxes.

**Figure S2.** Patient profiles of connected device use.

On the x-axis is the time scale of the search for each patient. The vertical black line represents the start of intervention at M0. On the y-axis are the different parameters measured by the connected devices (sleep, "freq": heart rate, "poids": weight, sao2, "Vems": fev1), and at the top of the y-axis there are the exacerbations ("ex") noted in the medical record. The gray areas that surround the exacerbations represent a period of +/- 3 days around the declaration of exacerbation to study the occurrence of an alert in this time window.


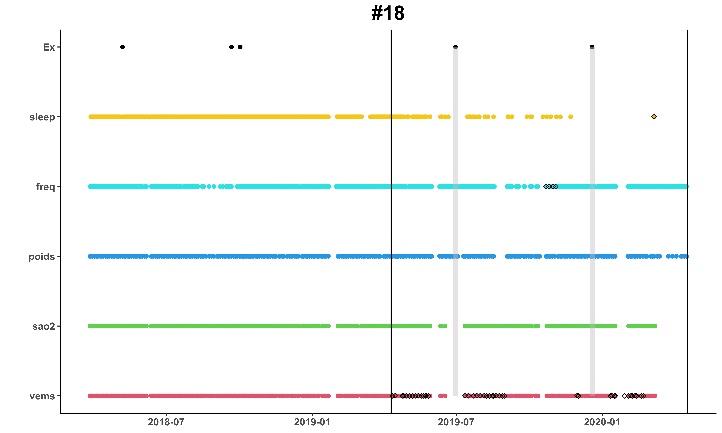
Profile 1 : Patients very compliant before M0 and during the intervention on all parameters


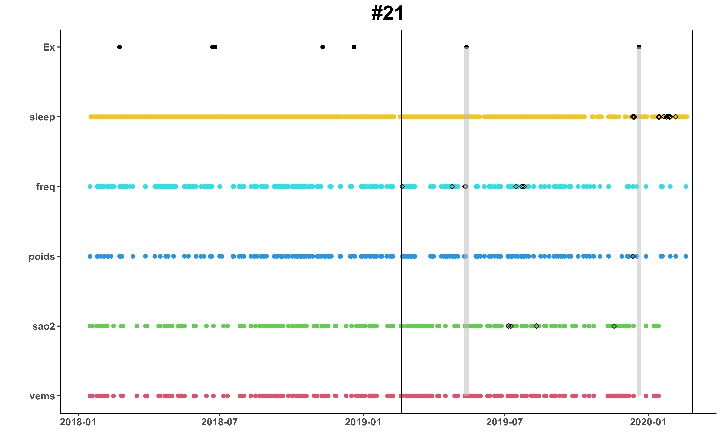

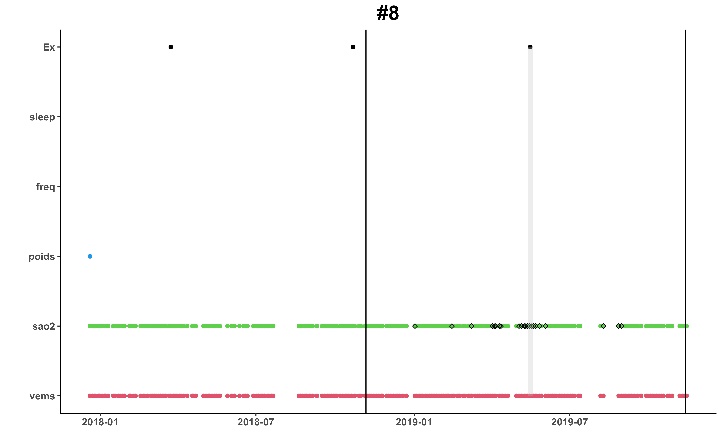


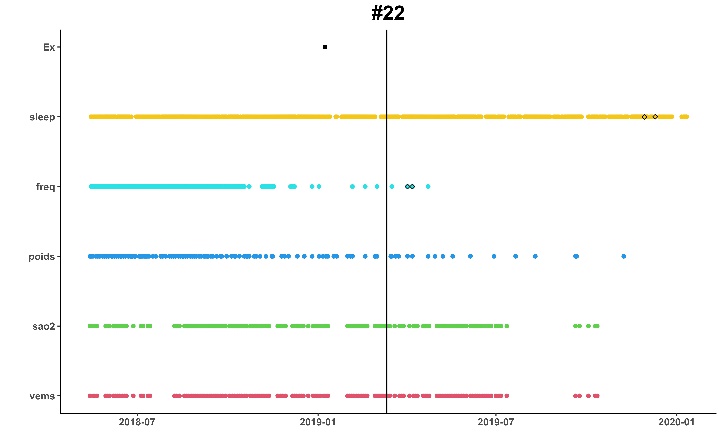

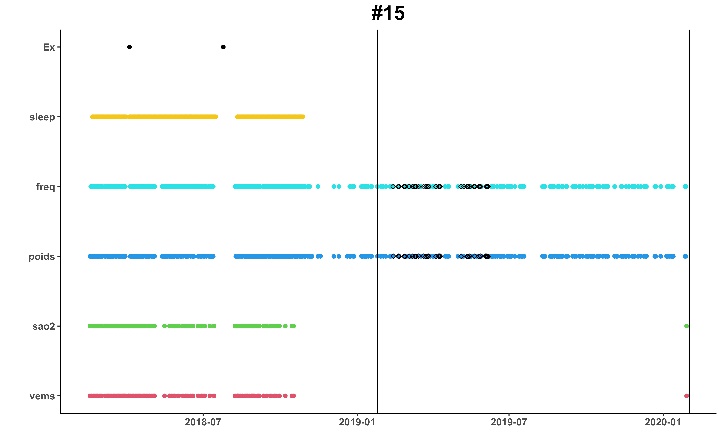
Profile 2 : Patients very compliant before M0 and on a selection of parameters during the intervention


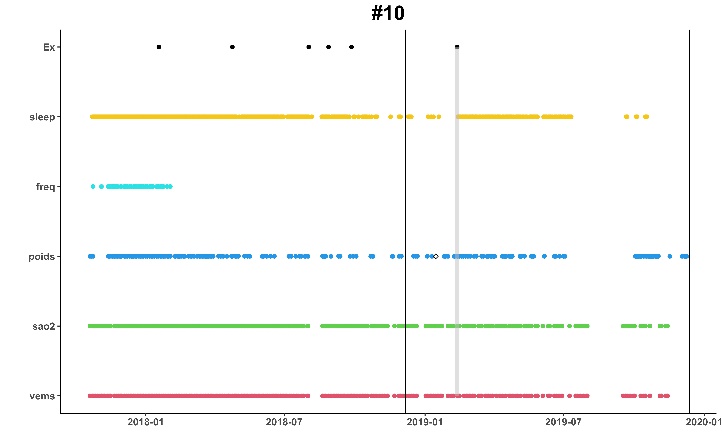

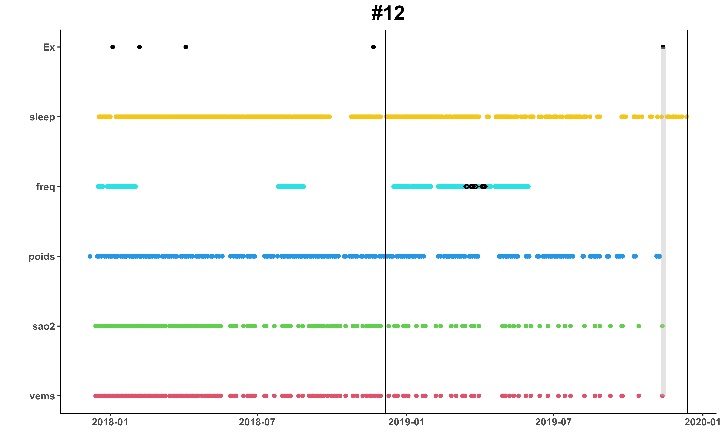


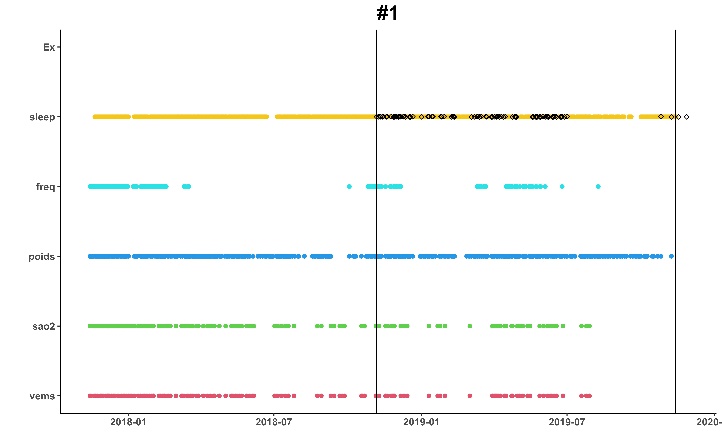


Profile 3 : Patients very compliant before M0 and moderately compliant during the intervention for any parameters


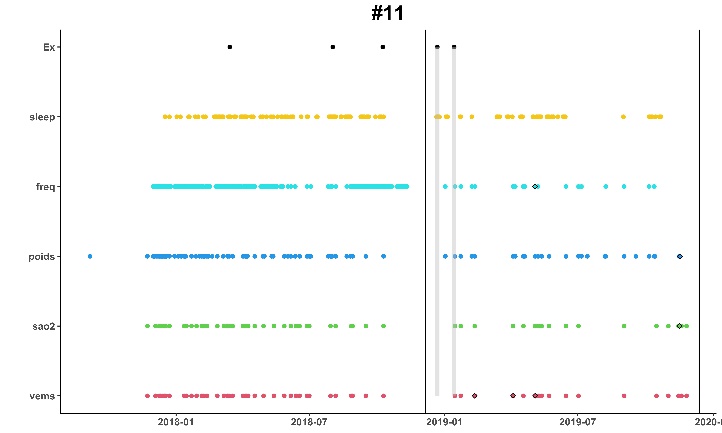

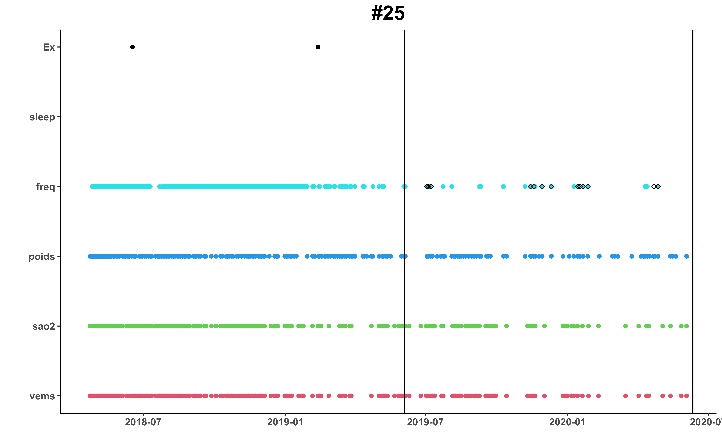


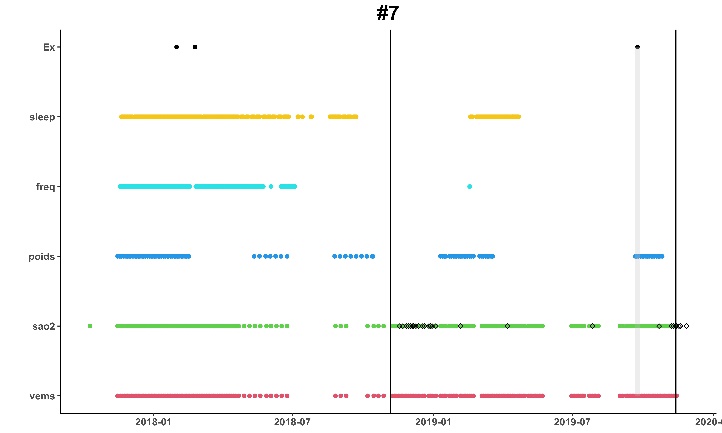


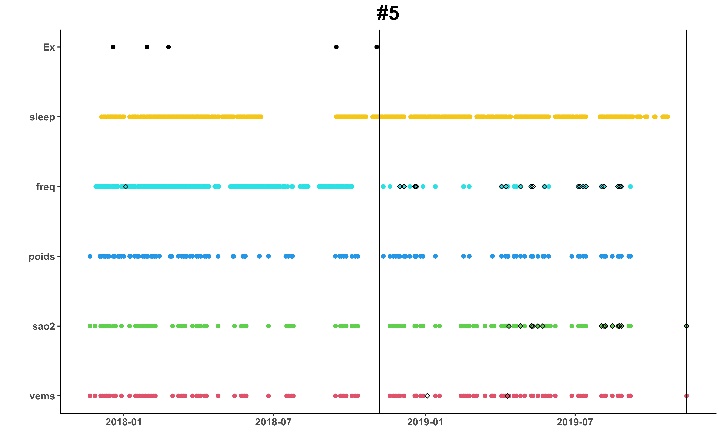


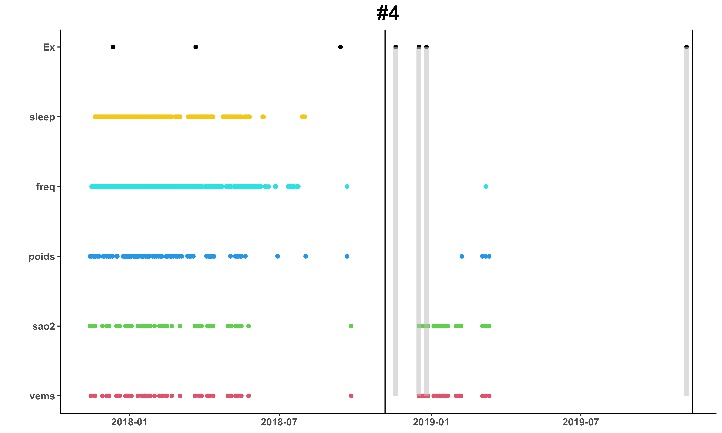
Profile 4 : Patients compliant before M0 and not compliant during the intervention


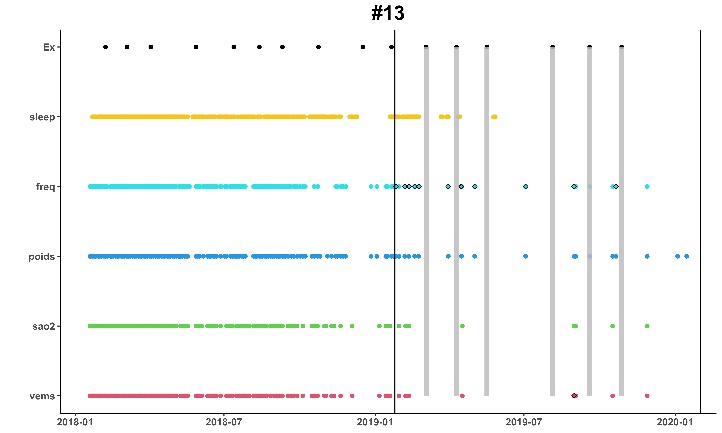

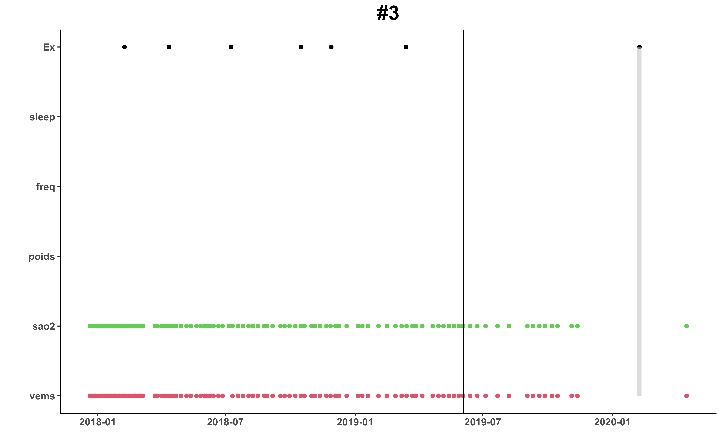


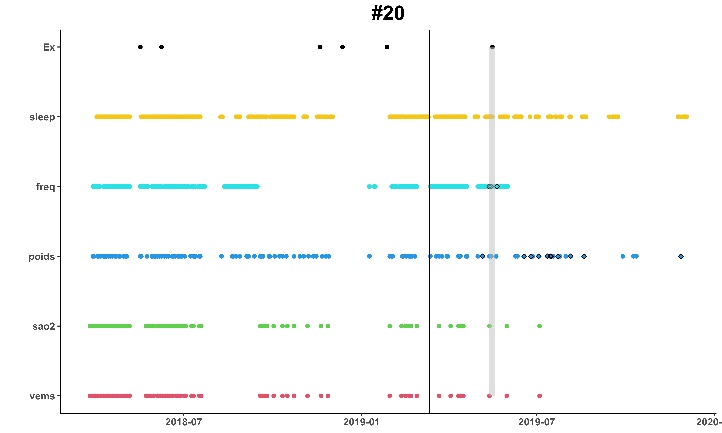

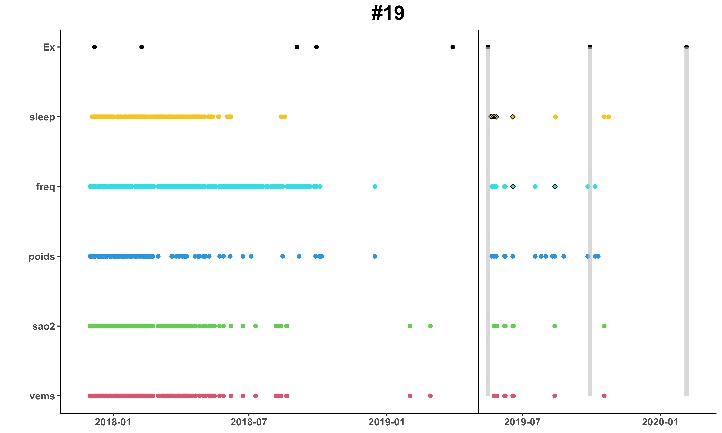

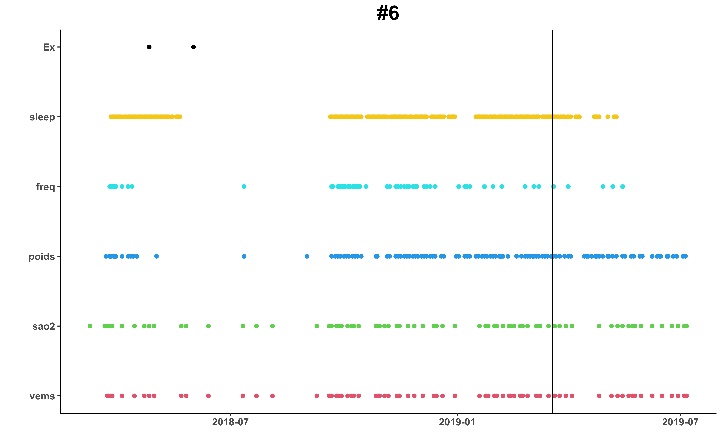


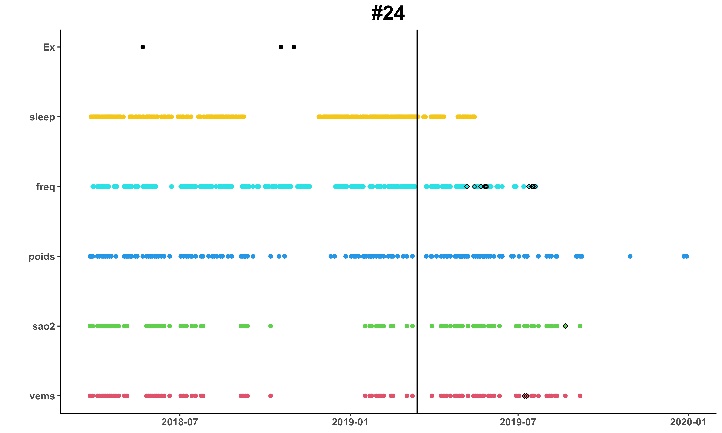


Profile 5 : Patients neither compliant before M0 nor during the intervention


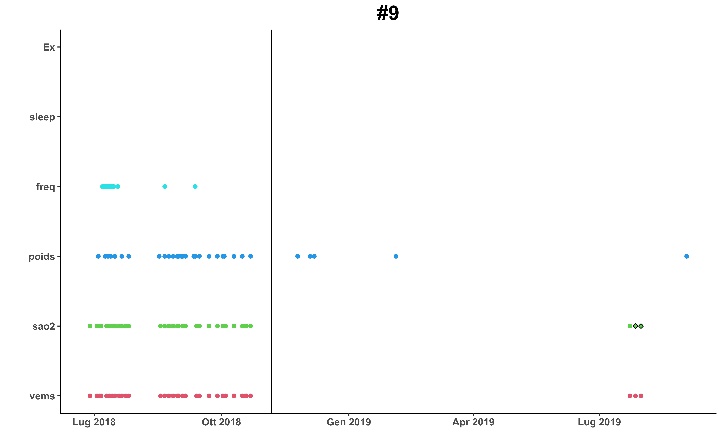

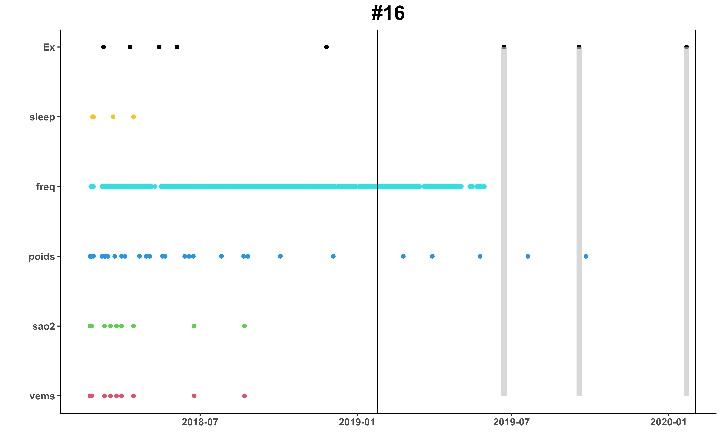

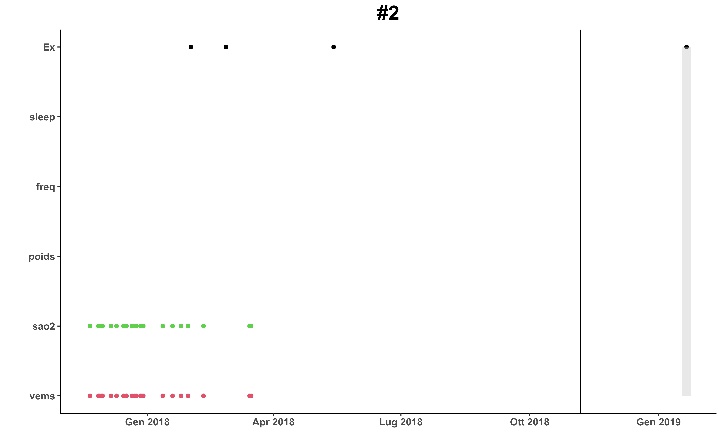


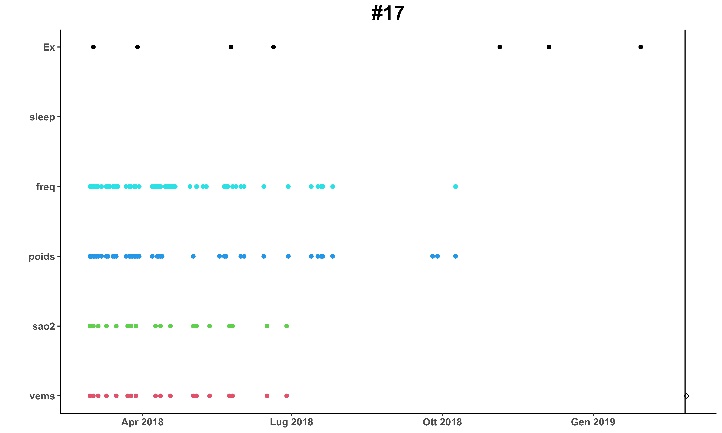

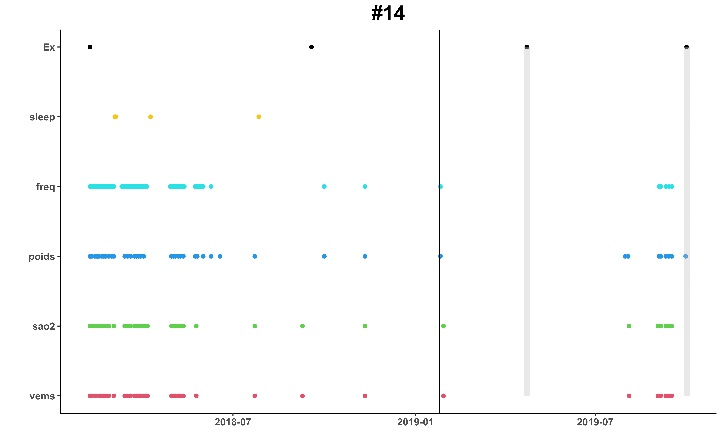

Supplement: Multimedia Appendix 2 [file formative_v8i1e51753_app2.docx]
